# 36H: A Novel Potent Inhibitor for Antimelanogenesis

**DOI:** 10.1155/2018/6354972

**Published:** 2018-02-04

**Authors:** Li-Ching Lin, Chung-Yi Chen, Chia-Hung Kuo, Yun-Sheng Lin, Byeong Hee Hwang, Tina Kaiting Wang, Yueh-Hsiung Kuo, Hui-Min David Wang

**Affiliations:** ^1^Department of Fragrance and Cosmetic Science, Kaohsiung Medical University, Kaohsiung 807, Taiwan; ^2^School of Medical and Health Sciences, Fooyin University, Kaohsiung 831, Taiwan; ^3^Department of Seafood Science, National Kaohsiung Marine University, Kaohsiung 811, Taiwan; ^4^Department of Biological Science and Technology, Meiho University, Pingtung 912, Taiwan; ^5^Division of Bioengineering, Incheon National University, Incheon, Republic of Korea; ^6^University of British Columbia, Department of Integrated Sciences for Physiology and Behavioural Neuroscience, Vancouver, BC, Canada; ^7^Department of Neuroscience, Washington State University, Pullman, WA, USA; ^8^Department of Chinese Pharmaceutical Sciences and Chinese Medicine Resources, China Medical University, Taichung 404, Taiwan; ^9^Department of Biotechnology, Asia University, Taichung 413, Taiwan; ^10^Graduate Institute of Biomedical Engineering, National Chung Hsing University, Taichung 402, Taiwan; ^11^Center for Stem Cell Research, Kaohsiung Medical University, Kaohsiung 807, Taiwan; ^12^Department of Medical Laboratory Science and Biotechnology, China Medical University, Taichung 404, Taiwan

## Abstract

*N*-Hydroxycinnamoylphenalkylamides (36H) exhibited both antioxidation and antityrosinase abilities. The compound was studied for its antioxidative properties, using a 1,1-diphenyl-2-picrylhydrazul- (DPPH-) scavenging test, a ferric ion-reducing antioxidant power assay (FRAP) assessment, and a metal-chelating power assay. The results showed that 36H had antioxidative capabilities in the DPPH-scavenging and ferric-reducing power examinations but the chelating power assay did not demonstrate antioxidative capability. 36H was also measured for tyrosinase inhibitory activity applying various species platforms, including in vitro mushroom, B16F10 mouse melanoma, and human melanocyte cells. In terms of in vitro mushroom tyrosinase suppression, 36H restrained the melanogenesis processes. It is assumed that 36H blocked the tyrosinase active site as a competitive inhibitor for mushroom tyrosinase, hence not decreasing the human normal melanocyte cellular viability. A quantitative real-time polymerase chain reaction (qRT-PCR) and western blot discovered that 36H downregulated melanogenesis-related RNA and proteins, including pigment production (MITF, tyrosinase, TRP-1, and TRP-2), melanosome maturation (Rab27a), and melanosome transportation (Myo5a, MLPH and Mreg). Overall, 36H displayed the biofunctions of antioxidation and melanin suppression, so there was a possibility for its application as a food additive or a skin-whitening agent.

## 1. Introduction

The human skin is the largest organ in the human body; it maintains bodily functions and prevents the loss of water, electrolytes, and biomolecules. The skin is composed of the epidermis, the dermis, and the hypodermis. The epidermis is further divided into five separated layers: the stratum corneum, the stratum lucidum, the stratum granulosum, the stratum spinosum, and the stratum basale [1]. The base layer of the epidermis, which is connected to the dermis, is the stratum basale that contains melanocytes. Melanocytes have dendrites to transfer melanin to keratinocytes, which determine the surface of the skin color due to the melanin content within these keratinocytes [2].

Free radicals are composed of an atom or a group of atoms that have one or more unpaired electrons. They are involved in physiological, metabolic, and immune reactions and signal transfer functions. Although they are a part of the normal healthy biochemical processes in the body, they also are responsible for damage. Reactive oxygen species (ROS) and various free radicals are stimulated by the generation of superoxide anions or hydrogen peroxides. This can cause confused messaging, damage to cell membranes, and damage to ion cell communications due to lipid peroxidation. This affects intracellular molecules that feature SH groups and acting proteins and DNA [3]. The amount of ROS is controlled by the self-defense systems that are antioxidant-mediated in their normal state, such as antioxidants. These include vitamin C, vitamin E, or glutathione [4], which scavenge free radicals to prevent cellular damage. However, they disrupt the balance of the cellular oxidation state which results in too many ROS. Previous studies have shown that many degenerative diseases, such as aging and cancer, are caused by active oxygen species or free radicals. The antioxidant properties suggest that the peroxidation of lipid within melanocyte membranes increases the content of intracellular glutathione, which may account for the depigmentation [5, 6].

Darkening of the skin, eyes, and hair is caused by the synthesis of melanin, which is a pigmented biopolymer that is synthesized in the melanosome of melanocytes. Melanin is a protective mechanism against damage by UV light [7]. Congenital genes can affect the skin color, but UV radiation induction, inflammation, and hormonal changes cause melanocytes to synthesize more melanin. Nevertheless, the overproduction of melanin can induce pigment disorders, such as melasma, senile lentigo, freckles, and hyperpigmentation [8]. These are usually treated using cosmetics or pharmaceutical ingredients that contain skin-whitening components. Even though these elements are from natural sources, only some agents are used in cosmetics or medicines because of safety concerns or the concerns of whitening effectiveness. Melanogenesis involves the binding of the *α*-melanocyte-stimulating hormone to the melanocortin-1, which increases cyclic adenosine monophosphate (cAMP) and activates microphthalmia-associated transcription factors (MITF) [9]. MITF upregulates the expression of a melanogenic enzyme, tyrosinase, which serves an important function in the hydroxylation of L-tyrosine to dihydroxyphenylalanine (L-DOPA) and the oxidation of L-DOPA to dopaquinone [10]. Medical and cosmetic treatments are increasingly using tyrosinase activity inhibitors. Melanogenesis production and the transfer of melanosomes from melanocytes to keratinocytes are necessary for the coloration of skin [11]. Melanosomes are tethered to the actin cytoskeleton at the periphery of melanocytes via the tripartite complex formed by Ras-related protein Rab-27 (Rab27a), melanophilin (MLPH), and myosin Va (Myo5a). Rab27a is a membrane-bound protein involved in protein transportation and small GTPase-mediated signal transduction. Melanophilin forms a ternary complex with Rab27a in the GTP-bound site with Myo5a which is the motor protein. The visible mammal pigmentation in the hair and skin is according to this tri-protein complex to tether the pigment-producing organelles. If there is a deficiency in the complex protein, the connection between the F-actin and melanosome is broken down [12].

Within the positive therapeutic potential compounds, *N*-hydroxycinnamoylphenalkylamides (36H) are reported to have an inhibitory effect on the expression of matrix metalloproteinases- (MMP-) 9 in THP-1, a human leukemia monocytic cell line, which is stimulated by tumor necrosis factor-*α* (TNF-*α*) [7]. A previous study showed that the induced TNF-*α* expression of MMP-9 for both protein and mRNA levels was fully inhibited in a concentration-dependent manner (1–20 *μ*M). It has been found that 36H markedly suppressed nuclear factor-*κ* light polypeptide gene enhancer in B cell (NF-*κ*B) signaling as detected by the NF-*κ*B reporter gene assay but had no effect on the degradation of (nuclear factor of *κ* light polypeptide gene enhancer in B cell inhibitor *α* (I*κ*B*α*) or the translocation of NF-*κ*B. Chromatin immunoprecipitation data illustrated that the affiliation between MMP-9 and NF-*κ*B p65 subunit (p65) promoter gene was entirely negated by 36H and the phosphorylation of p65 was not influenced. In general, a previous report demonstrated that 36H suppressed MMP-9 secretion by the nuclear-targeted downregulation of NF-*κ*B pathway mechanisms in THP-1. 36H is an analogue of caffeic acid phenethyl ester (CAPE), which is a known suppressor of cancer metastasis and invasion [14]. The antioxidant activities and free radical-scavenging of some CAPE analogues have been studied, and the results of those studies prompted this study to determine whether 36H reduced tyrosinase activity and downregulated melanogenesis-related RNA and proteins. This study finds that 36H is a potential and positive antioxidant and a skin-whitening agent.

## 2. Materials and Methods

### 2.1. Reagents and Materials

Dimethyl sulfoxide (DMSO) and L-tyrosine were obtained from Sigma-Aldrich Chemical Inc. (St. Louis, MO, USA). Dulbecco's modified Eagle's medium (DMEM) and fetal bovine serum (FBS) were purchased from Gibco-BRL (Gaithersburg, MD, USA). All reagents and alternative buffers were acquired at the highest commercial purity.

### 2.2. Chemical Synthesis of 36H

**Figure 1:**
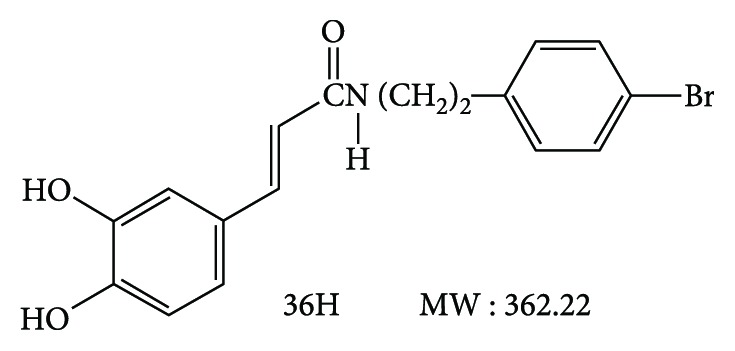
The chemical structure of *N*-hydroxycinnamoylphenalkylamides (36H).

36H was provided by Professor Yueh-Hsiung Kuo, who used the subsequent methods from amide-binding coupling to obtain the compounds. Benzotriazol-1-yloxytris (dimethylamino)-phosphonium hexafluorophosphate (BOP) was combined with a mixture of R2-NH2, R1-COOH, and triethylamine (Et3N) in dimethylformamide (DMF). The reaction solution was mixed for 30 min at 0°C and then blended at 25°C for 2 hrs. When the chemical liquid had been evaporated, the residue was divided between H_2_O and ethyl acetate (AcOEt). The residue was then filtered and purified using column chromatography with an eluting solution (AcOEt–CH_2_Cl_2_, 1 : 1, *v*/*v*) on silica gel (70–230 and 230–400 mesh, Merck 7734). The products were recrystallized from AcOEt to obtain the best and most ideal crystals. A Finnigan TSQ-46C mass spectrometer was used for the electron impact mass spectrometry (EIMS). 1H and 13C NMR spectra were obtained using a Bruker Avance 500 spectrometer. A Nicolet Magna-IR 550 spectrophotometer recorded the IR spectra. 36H was initially dissolved in DMSO solution before the following studies. For each assay, DMSO with a constant and final concentration of 0.2% (*v*/*v*) was used. This compound has not been known as an antioxidant or skin whitener [7, 13], and the structure is shown in Figure 1. Samples were dissolved in DMSO, and various concentrations were prepared with a final DMSO concentration less than 0.5%.

### 2.3. Assay of DPPH Radical-Scavenging Capacity

DPPH is 2,2-diphenyl-1-picrylhydrazyl, which becomes dark purple when it has a free radical. When the antioxidant scavenges the radical from DPPH˙, it reduces DPPH˙ to a stable DPPH, which has a light-yellow color [14]. Various dosages of the 36H (2 *μ*L in total volume) were combined in 98 *μ*L of DPPH (60 *μ*M) mixture, and the plate was examined using a 517 nm enzyme-linked immunosorbent assay (ELISA) spectroscopic reader. L-ascorbic acid was used as the positive control. The ratios of residual DPPH were mapped beside the sample to determine the amount of antioxidant that decreases the former DPPH concentration. The scavenging property (%) was determined as

(1) $$\begin{array}{c} Scavenging\;activity\;\left(\%\right)=\frac{\left({OD}_{control}-{OD}_{sample}\right)}{{OD}_{control}}\times 100\%. \end{array}$$

### 2.4. Assay of Reducing Power

The ferric ion-reducing antioxidant power assay (FRAP) is often applied to evaluate the antioxidative capability of beverages, foods, fruits, and nutritional supplements, including flavonoids or polyphenols. Various dosages of 36H were introduced into a 67 mM phosphate buffer mixture (0.085 mL, pH 6.8) and 20% potassium ferricyanide [K_3_Fe(CN)_6_, 2.5 *μ*L). The solution reacted for 20 min at 50°C and then trichloroacetic acid (10%, 0.16 mL) was added into the solution, before it was centrifuged at 3000*g* for 10 min [14]. The solution supernatant (75 *μ*L) was combined with 2% FeCl_3_ (25 *μ*L), and then an ELISA spectroscopic reader measured the resulting absorbance at 700 nm, for a 96-well plate. Butylated hydroxyanisole (BHA) was employed as the positive control. The greater the reductive qualification, the stronger is the absorbance.

### 2.5. Assay of Metal-Chelating Activity

Chlorophyll's ion-chelating ferrous potential was measured using the technique of Chen et al. [14]. In brief, specific dosages of the samples were dissolved into DMSO and were introduced into a mixture of FeCl_2_·4H_2_O (2.0 mM, 0.05 mL). The addition of ferrozine (5 mM, 0.2 mL) initiated the reaction when the solution was forcefully shaken and then left for 10 min at 25°C. When the reaction had reached equilibrium, the absorbance values for the solution were determined at 560 nm using a blank vehicle. EDTA was used as the positive control. The calculation formula for the chelating activity was identical to (1).

### 2.6. Assay of the Mushroom Tyrosinase Activity

Both the mushroom tyrosinase activity suppression and the cellular tyrosinase inhibition were spectrophotometrically determined using the previous method, with minor modifications [2]. Kojic acid was used as the positive control for the tyrosinase activity assay. The materials were first dissolved in aqueous DMSO and cultured in L-tyrosine (2.5 mg/mL) with phosphate buffer (50 mm, pH 6.8). All models used DMSO, which is irrelevant to tyrosinase activity when DMSO constitutes <0.5% of the total volume. Subsequently, 25 U/mL of mushroom tyrosinase with an identical buffer was then added, and the solution was incubated at 37°C for 30 min. An ELISA spectroscopic reader was used to conduct the absorbance measurements for the assays at 475 nm, using 96-well microplates (Molecular Devices, CA, USA).

### 2.7. Human Melanocyte Cell (HMC) Cultures

Primary human epidermal melanocytes from neonatal foreskin were acquired from Cascade Biologics. This was also applied by Wang et al. [2]. It was cultured in Medium 254 (M-254-500; Cascade Biologics, Portland, OR, USA) and enhanced with human melanocyte growth supplement (HMGS, cat. number S-002-5). Medium 254 is a basal medium with some nonessential and essential vitamins, organic compounds, amino acids, inorganic salts, and trace minerals. The human melanocyte growth supplement included fetal bovine serum, bovine insulin, bovine pituitary extract, bovine transferrin, hydrocortisone, basic fibroblast growth factor, phorbol 12-myristate 13-acetate, and heparin. All cells were incubated at 37°C in a humidified incubator with 5% CO_2_ atmosphere.

### 2.8. Determination of Cell Viability of HMC

Cell viability was determined using a MTT assay [3]. When a yellow tetrazole is combined with the mitochondrial dehydrogenase (ubiquinone) in living cells, the tetrazolium rings of MTT cleave and reduce to purple formazan. The cells were seeded at a density of 9 × 10^3^ cells/well in 96-well plates. When the cells had been cultured for a day, the medium was changed, and different concentrations of 36H were added into a final medium volume of 100 *μ*L. After these had incubated for 48 hrs, the medium was substituted with a fresh medium (100 *μ*L), including MTT (0.5 mg/mL). The plate was then cultured in an incubator at 37°C for 2 hrs with 5% CO_2_. DMSO (100 *μ*L) was then added to each well to dissolve the purple formazan crystals. To ensure that the dishes achieved maximum dissolution, the dishes were gently shaken and mixed in the dark for 10 min and measured at 595 nm. Cell growth was calculated as

(2) $$\begin{array}{c} Cell\;growth\left(\%\right)=\frac{\left(A_{sample}-A_{blank}\right)}{\left(A_{control}-A_{blank}\right)}\times 100\%. \end{array}$$

### 2.9. Measurement of Tyrosinase Activity from HMC

To determine the tyrosinase activity in human melanocyte cells (HMC) (5 × 10^4^ cells per well), they were positioned in 12-well plates in 1000 *μ*L of medium with different dosages of 36H and cultured for 48 hrs [2]. PBS buffer was used to wash the sample-treated cells, and these were then lysed with 1% Triton X-100/PBS. The enzyme extract was then collected from the resulting cellular lysate and combined with 50 *μ*L of 2 mM L-tyrosine. The extracts were then incubated for 3 hrs at 37°C in darkness. A spectrophotometer was then used to determine their absorbance at 490 nm.

### 2.10. Determination of the Melanin Content in HMC

With some minor modifications, the previous technique was also used for this experiment [2]. Cell pellets were dissolved in 2.0 N NaOH with 10% DMSO and heated to 90°C for 1 hr. The suspensions were separated by centrifuging for 10 min at 10,000*g*. Using a spectrophotometer, the melanin content was determined via the data produced at an absorbance level of 475 nm.

### 2.11. Quantitative Real-Time Polymerase Chain Reaction

For the qRT-PCR, a 20 *μ*L reaction contained a 0.4 mL mixture of two reverse transcriptases: 10 *μ*L of 2 × QuantiTect SYBR Green Master Mix (QIAGEN, Valencia, CA, USA) with the hot start Taq polymerase, 0.8 mL of primers, and 0.5 mL (10 ng/mL) of template. The primer sequences are listed in Table 1. The StepOnePlus™ System was used for all real-time PCR assays. The reaction was completed by performing the RT reaction at 42°C for 20 min and then activating the FastStart Taq DNA polymerase at 95°C for 5 min. This was then amplified for 40 or 50 cycles at 95°C for 5 sec for denaturation, annealing, and acquisition at 60°C for 5 sec. It was finally elongated at 72°C for 15 sec. Fluorescence can also be measured after the elongation phase, but for this experiment, it was measured after the annealing phase. With a 96-well plate, 9 *μ*L of the lysate was added to 11 *μ*L of the reaction mix, which consisted of 10.6 *μ*L MasterMix from the Eurogentec qPCR core kit for SYBR Green I (1.4 *μ*L of 50 mM MgCl_2_, 2 *μ*L 10x reaction buffer, 0.8 *μ*L of 5 mM dNTP mix, 0.6 *μ*L SYBR Green I, 5.7 *μ*L of nuclease-free water, and 0.1 *μ*L of hot GoldStar Taq polymerase), 0.1 *μ*L MS2 RNA, 0.1 *μ*L 10x diluted RNAse inhibitor, and 0.1 *μ*L of the REV primer (100 *μ*M), as well as the MS2 FWD for SG-PERT assays on the ABI 7300 qPCR system. The qRT-PCR reaction, activation of the hot GoldStar Taq enzyme, 40-cycle amplification, annealing and acquisition, denaturation at 95°C, and the elongation at 72°C used the ABI 7300 instrument. To prepare the assay, all of the reagents were kept either on a cooling block or on ice. Duplicate SG-PERT reactions were performed on each lysate sample. Using qPCR software and instruments, an ABI 7300 with its threshold determined manually and a LightCycler® 480 with its maximum second-derivative method, generated the cycle of quantification (Cq) values. Using the same software for both instruments, the melting peaks were also automatically calculated.

### 2.12. Western Blotting

A total of 1 × 10^5^ cells were treated with sample groups or the blank vehicle control for two days. The cells were harvested and lysed with lysis buffer (50 mM Tris-HCl, pH 7.5, 137 mM sodium chloride, 50 mM sodium fluoride, 10 mM sodium pyrophosphate, 20 mM *β*-glycerophosphate, 1 mM phenylmethylsulfonyl fluoride, 1 mM EDTA, 10% glycerol, 1% Nonidet P-40, 2 *μ*M leupeptin, 0.1 mM sodium orthovanadate, and 2 μg/mL aprotinin) [14]. Lysates were centrifuged at 20,000 ×g for 30 min, and the protein concentrations within the supernatant solution were determined with a bicinchoninic acid (BCA) protein assay kit (Pierce, Rockford, IL, USA). Equal amounts of protein were separated by sodium dodecyl sulfate-polyacrylamide gel electrophoresis (SDS-PAGE) and then electrotransferred to a nitrocellulose membrane (PALL Life Science, Ann Arbor, MI, USA). The membrane was blocked for 1 hr with 5% nonfat milk in PBS-T buffer (PBS containing 0.1% Tween 20). The transfer film was carefully removed from the wet transfer tank and semidry transfer slot and placed in a box that had 5% skimmed milk in 1x TBST for 1 hr at room temperature. It was then gently washed with 1x TBST to remove traces of the skimmed milk. The membrane was then incubated with respective primary antibodies. In each case, the membranes were incubated with horseradish peroxidase-conjugated antirabbit or mouse antibody and then treated with ECL detection reagents (PerkinElmer, ECL1 : ECL2 = 1 : 1). A mini-sized chemiluminescent imaging system from Life Science was used for measurement to detect the bands [14].

### 2.13. Statistical Analysis

Three of each concentration for the standard and the samples were used. Using Student's *t*-test, the results were statistically compared and were expressed using the average of the mean values ± standard deviation (SD).

## 3. Results

### 3.1. DPPH Free Radical-Scavenging Activity

ROS can generate lipid peroxides, DNA damage, protein expression, tissue aging, and cancer genesis when the balance between free radicals and inherent antioxidants is disturbed. Free radical chain reactions are blocked by neutralizing the electron donations or acceptances and chelating free lone pairs. Reducing oxidative stress from intrinsic and extrinsic facets is one way to increase health. This study examined antioxidative properties, by determining the ferric-reducing power, the DPPH free radical-scavenging capacity, and the metal-chelating power. The increased generation and accumulation of ROS produces lipid oxidation in food and cosmetics and natural antioxidative activity. In particular, free radical-scavenging properties are vital in functional nutrition additives and skin care products.

The first oxidation inhibitory assay was the DPPH free radical-scavenging test. This is a simple and economical experimental platform, in which antioxidants act to prevent oxidation products. Antioxidants change the color of the stable radical DPPH reagent from purple to the light yellow of diphenyl-picrylhydrazine. To determine the antioxidative properties of 36H, a 100 *μ*M dose was applied to determine the scavenging ability.  Table 2 shows that 36H exhibited excellent free radical scavenging ability and scavenges 60% of the DPPH free radical and vitamin C at the same concentration (100 *μ*M) scavenges 85.3%.

### 3.2. Ferric Reducing Antioxidant Power

The second oxidation inhibitory assay is the ferric-reducing power test, which is a common and reliable way to measure the synthesis of Fe(III)–ferricyanide complex. A functional agent reduces Fe^3+^/ferricyanide complexes to the ferrous form. This combination had a blue color at 700 nm, because K_4_Fe(CN)_6_ reacted with Fe^3+^ to form Fe[Fe(CN)_6_]_3_. In the experiment, the color of the solution changed from light yellow to different shades of blue and green, depending on the reducing power of the target compound.  Table 2 presents that BHA at 100 *μ*M had a reducing power value of 0.95 and 36H at 100 *μ*M had a reducing power value of 0.85 compared to BHA. Therefore, 36H was shown to have good ferric-reducing antioxidant power.

### 3.3. Ferrous Ion-Chelating Capacity

The chelating activity for ferrous ion of 36H is shown in Table 2. EDTA (100 *μ*M) was used as a positive control. Fe^2+^ and ferrozine form complexes quantitatively. The formation of reagent complexes is often disrupted, because of chelating agents, which results in a reduction in the red color of the complex. 36H at a dosage of 10 *μ*M had no significant effect on Fe^2+^-scavenging capacity, and at the concentration of 100 *μ*M, it presented 43.2% inhibition. The positive control, EDTA, had approximately 80% ion-chelating capacity at 100 *μ*M.

### 3.4. Effect of 36H on Mushroom Tyrosinase Activity

**Figure 2:**
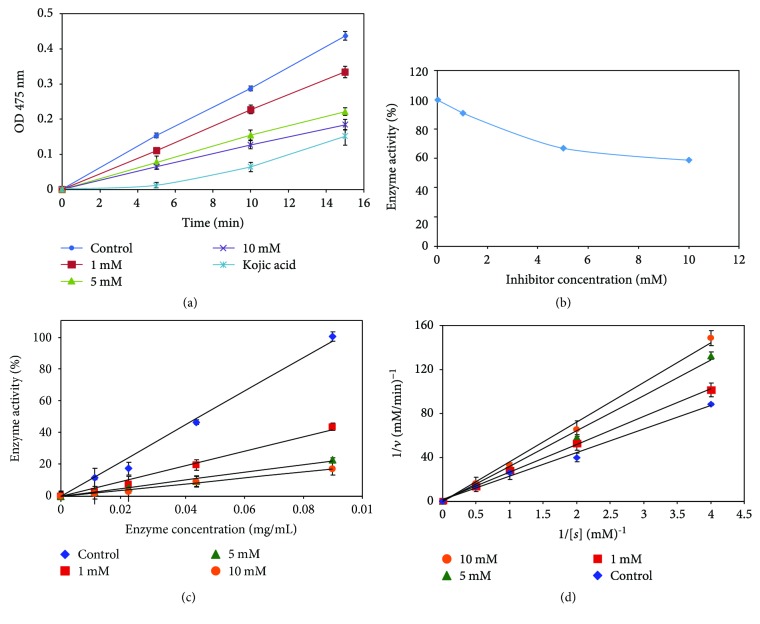
Inhibitory enzyme kinetic effects of 36H on mushroom tyrosinase. (a) 36H at different concentrations (1, 5, and 10 mM) affects mushroom tyrosinase activity. The concentration of positive control, Kojic acid, is 10 mM. Data are representative of 3 experiments. (b) The inhibition of mushroom tyrosinase enzyme activity of 10 mM. (c) Inhibition effects of 36H at different concentrations (1, 5, and 10 mM) on the activity of mushroom tyrosinase for the oxidation of L-tyrosine. (d) Inhibitory effect of 36H at different concentrations (10, 50, and 100 mM) on mushroom tyrosinase. The data for Lineweaver-Burk plots were obtained as mean values of three independent assays with various concentrations of L-tyrosine (0.125, 0.25, 0.5, 1, and 2 mM) as the substrate.

The inhibition of tyrosinase activity was described in numerous reports, most of which used mushroom tyrosinase as the model. The advantages are being well-developed by scientists, easy procedures, low cost, rapid pigmentation process, similar structural and functional characteristics as mammals, and convenience in observing melanin development. The inhibition of mushroom tyrosinase activity by 36H was studied, and the inhibitory ability had a positive correlation with the concentration of 36H (Figure 2(a)). In the fifteenth minute, it was seen that 36H at different concentrations (1, 5, and 10 mM) compared to the control reduces the OD values. The enzyme activity decreases by about 10% at 1 mM, 30% at 5 mM, and 40% at 10 mM, compared to the vehicle control (Figure 2(b)). The relationship between tyrosinase activity and its concentration in 36H was studied. Different concentrations of the inhibitor give a group of lines that all pass through the origin. The inhibition of tyrosinase activity by 36H had no effect on the amount of enzyme, which illustrated that 36H was a reversible inhibitor (Figure 2(c)). The type of inhibition on tyrosinase by 36H was confirmed using a Lineweaver-Burk double-reciprocal plot. The plots of 1/*v* versus 1/[*s*] gave a family of straight lines through the origin, which presented that 36H was a competitive complex. The kinetics of the enzyme are presented in Figure 2(d).

### 3.5. The Effect of Cell Viability, Cellular Tyrosinase, and Melanin Content on 36H in HMC

**Figure 3:**
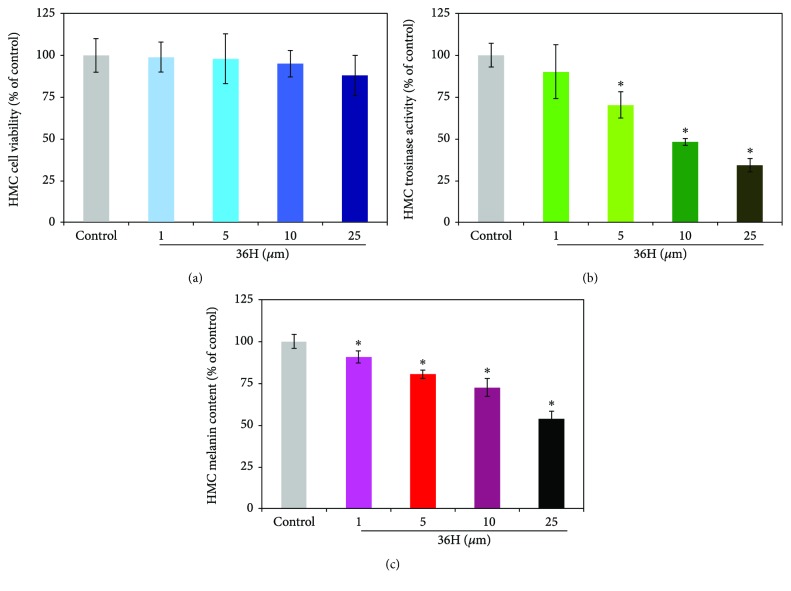
The inhibitory effect of 36H in HMC. (a) The impact of 36H at different concentrations (1, 5, 10, and 25 *μ*M) to human melanocyte cell viabilities. (b) The tyrosinase activity of the human melanocyte cell treated with various concentrations of 36H. (c) The melanin content of the human melanocyte cell treated with various concentrations of 36H. Data are shown as mean ± SD; *n* = 3; ^∗^*P* < 0.005, compared with the control groups.

As a potent skin-lightening compound, the component should be harmless, without undesirable cytotoxic side effects. There are some well-known melanin synthesis inhibitors, including kojic acid, arbutin, PTU, or hydroquinone, that are being utilized globally as cosmetic ingredients at present. We also discovered that these melanogenic inhibitors might induce human skin tumorigenicity at high concentration doses or frequent use [2]. HMC was cultured in 36H for 48 hrs at various concentrations of 1, 5, 10, and 25 *μ*M, and the cell viability was determined to show low cellular toxicities in Figure 3(a). When considering the agent for therapeutic or cosmetic usage in human beings, we found that the cytotoxic consequences on human dermal cellular viabilities were insignificant. To understand the inhibitory effect of 36H on melanogenesis, we assessed intracellular tyrosinase activity in HMC. The results for tyrosinase activity in HMC showed that there was an obvious variation as the concentration increased, and the tyrosinase activity decreased for low cell toxicity. After treatment, the tyrosinase activity was reduced to 39 ± 2.2% at 25 *μ*M in a dose-dependent manner (Figure 3(b)). These results showed that 36H inhibited intracellular tyrosinase activity. The melanin assay results clearly showed that 36H reduces the melanin content in HMC in a dose-dependent manner (Figure 3(c)). The percentage of control represents the melanin content. After treatment, the melanin contents were 77% at 25 *μ*M, 84% at 10 *μ*M, 88% at 5 *μ*M, and 90% at 1 *μ*M. These results showed that 36H had a significant inhibitory effect on the synthesis of melanin in HMC at 25 *μ*M.

### 3.6. The Melanin Biosynthesis-Related mRNA and Proteins Were Influenced by 36H in HMC

**Figure 4:**
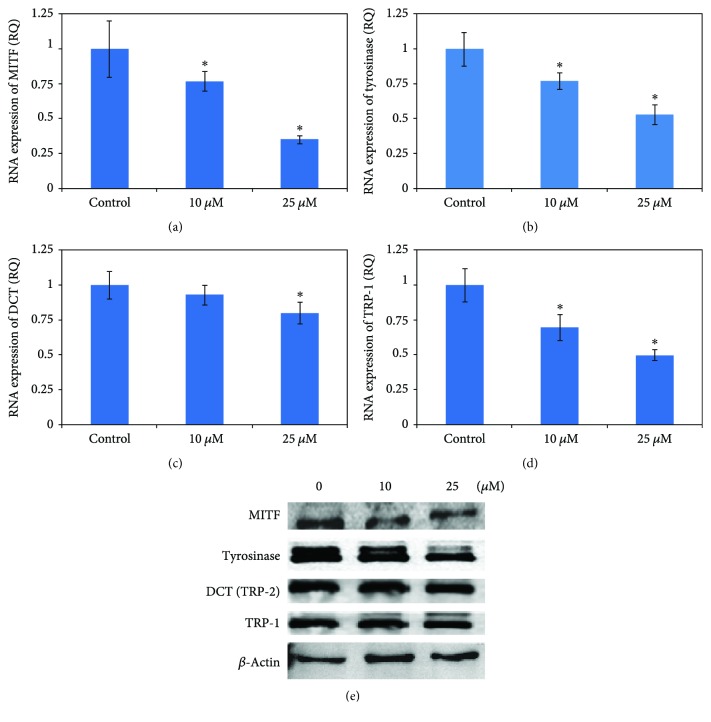
The RNA and protein expressions associated with melanin biosynthesis of human melanocyte cell treated with various concentrations (0, 10, and 25 *μ*M) of 36H. Data are representative of 3 experiments. ^∗^*P* < 0.05 as compared with the control.

Melanin biosynthesis occurs in melanosome, and the initial amino acid substrate is tyrosine. Tyrosine is firstly catalyzed to L-DOPA via tyrosinase, and using the same enzyme, tyrosinase, DOPA is catalyzed to dopaquinone. Dopaquinone is catalyzed by dopachrome tautomerase (tyrosinase-related protein-2, TRP-2), TRP-1, and tyrosinase to form eumelanin. In HMC, 36H downregulated the cellular melanogenesis-related RNAs and proteins as demonstrated in Figure 4.

### 3.7. Effect of 36H on the Melanosome Maturation

**Figure 5:**
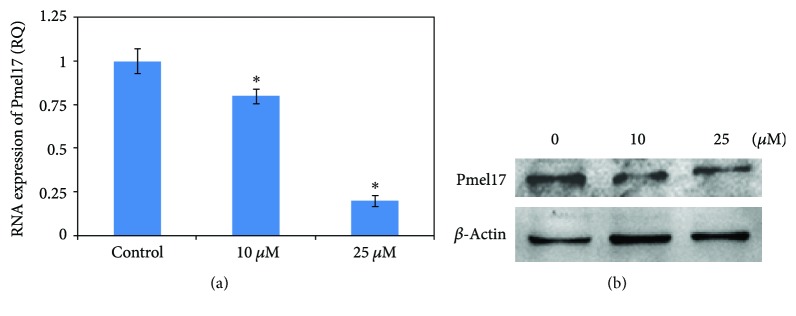
The RNA and protein expressions associated with melanosome maturation of human melanocyte cell treated with various concentrations (0, 10, and 25 *μ*M) of 36H. Data are representative of 3 experiments. ^∗^*P* < 0.05 as compared with the control.

The melanosome is an organelle that relies on melanin synthesis within melanocytes. The more melanosomes mature, the more melanin is formed. Therefore, melanosome maturation is important in the mechanism of skin whitening. Premelanosome protein 17 (Pmel17) is targeted to precursors of the pigment organelle, the melanosome, where it is proteolytically processed to several small fragments. Some of these fragments form nonpathological amyloids that assemble into sheets and form the striated pattern that underlies the melanosomal ultrastructure. This study demonstrated that 36H downregulated Pmel17 RNA and protein expression in HMC (Figure 5).

### 3.8. Effect of 36H on the Melanosome Transport

**Figure 6:**
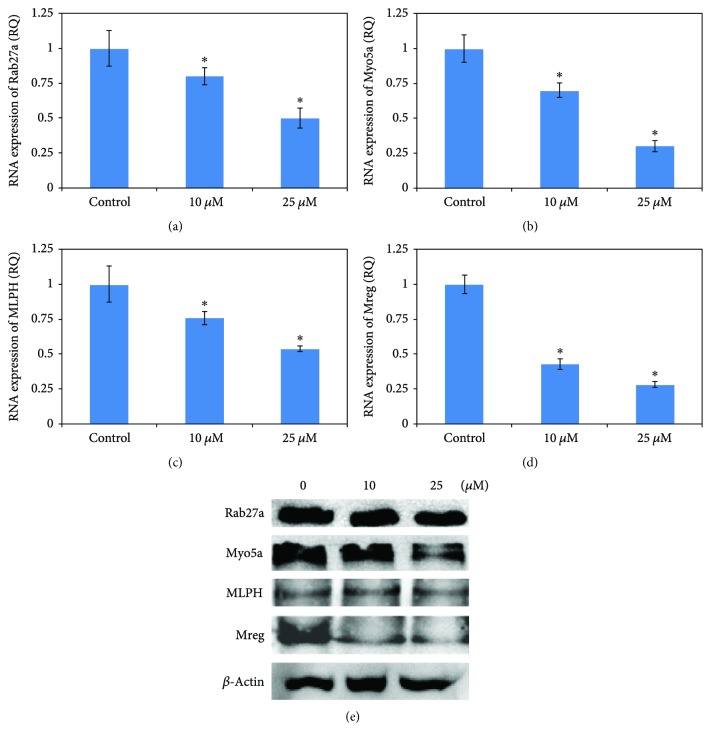
The RNA and protein expressions associated with the melanosome transport of human melanocyte cell treated with various concentrations (0, 10, and 25 *μ*M) of 36H. Data are representative of 3 experiments. ^∗^*P* < 0.05 as compared with the control.

Rab27a, MLPH, and Myo5a form a tri-protein complex to bind melanosomes at the melanocyte peripheries. In the process of melanosome transport, the ternary complex is the connection between actin cytoskeleton and melanosome. A lack of these proteins affects the transport, and melanoregulin (Mreg) drives melanosome transfer from melanocytes to keratinocytes via a regulated shedding mechanism. This study illustrated that 36H decreased the melanosome transport by affecting these related RNA and proteins (Figure 6).

## 4. Discussion

This study shows that antioxidants inhibit melanogenesis in two ways. In the melanin biosynthesis process [15], tyrosinase first transforms hydroxide tyrosine to DOPA, then oxidizes DOPA to dopaquinone [2]. Melanin scavenges free radicals to inhibit lipid peroxidation and protects the skin from UV damage, but melanin can also be deoxidized by an antioxidant. Therefore, melanin is called the radical sink [2, 16, 17]. A lack of melanin reduces the protection for the skin, so ROS stimulates melanocyte to produce more melanin [18]. Consequently, a good antioxidant can reduce tyrosinase activity and inhibit parts of the melanin synthesis. 36H had antioxidant properties in the DPPH free radical-scavenging ability and ferric-reducing power.

Before injecting the protein samples into SDS-PAGE, we normalized all protein levels. The protein normalization is a significant process applied to remove both experimental biological errors and artificial unexpected variabilities [2]. Genes encoded in DNA are transcribed into pre-messenger RNA (mRNA) by RNA polymerase, and then most organisms develop it using various posttranscriptional modification forms to generate the matured-mRNA, which is applied as a template for protein syntheses via ribosomes. The transcription unit is a stretched DNA to transcribe into RNA and transcripts mRNA which is provided as a template on the protein translation for the syntheses [3, 6]. In human beings, mRNA is in the cellular nucleus to be translocated across the nuclear membrane into the cytoplasm, which is the location where protein syntheses take place. The relationship between mRNA and protein is a complex network. The regulation of NDA transcriptions and translations could be differently changed. In cells, proteases degrade the functions of proteins into small amino acids or polypeptides. Due to intracellular breakdown, amino acids can be recycled for protein syntheses again. This mechanism cleans abnormal or damaged proteins and ones that are no longer needed to prevent unnecessary protein accumulations. Although we would consider that the amount of proteins decreased when the transcription of the encoding genes was reduced, there were other mechanisms regulating the protein abundances. For example, the protein's half-life might be increased due to a decreased rate of biodegradation. Another possibility was that the mRNA was more preferentially translated during the process. The human skin color is also affected by the melanosome regulative degradation autophagy in keratinocytes [19].

When keratinocytes are exposed to UV [20], they release *α*-melanocyte-stimulating hormone (*α*-MSH), adrenocorticotropic hormone (ACTH), and prostaglandins E_2_ (PGE_2_) [21]. These signaling molecules activate the downstream signaling pathway of adenylate cyclase through the melanocortin 1 receptor (Mc1R) on the membrane of the melanocyte to induce melanogenesis by enhancing MITF, tyrosinase, TRP-1, and TRP-2 [22] and through the IP3/DAG mechanism to activate the inactive-form tyrosinase to the active form. Our work demonstrated that 36H altered *MITF* RNA expression, but there was insignificant change in the amount of protein production. Tyrosinase affects melanin biosynthesis and TRP-2 and TRP-1 [23]. Dopachrome is catalyzed to 5,6-dihydroxyindole-2carboxylic acid by TRP-2, and 5,6-dihydroxyindole-2carboxylic acid is transferred to indole-5,6-quinone carboxylic acid via TRP-1 [24], which is then synthesized into eumelanin [16]. In mushroom tyrosinase and cellular tyrosinase assays, 36H downregulated tyrosinase activity. TRP-2 and TRP-1 were diminished at the RNA level, but there were insignificant differences in protein levels, compared to the control group. In melanosome maturation, Pmel17 is the precursor of melanosome. It is proteolyzed to fragments to form the striated pattern that underlies melanosomal ultrastructure [25]. Using a western blot, Pmel17 was shown to decrease in both RNA and protein expressions, which interrupted the maturation of melanosome.

**Figure 7:**
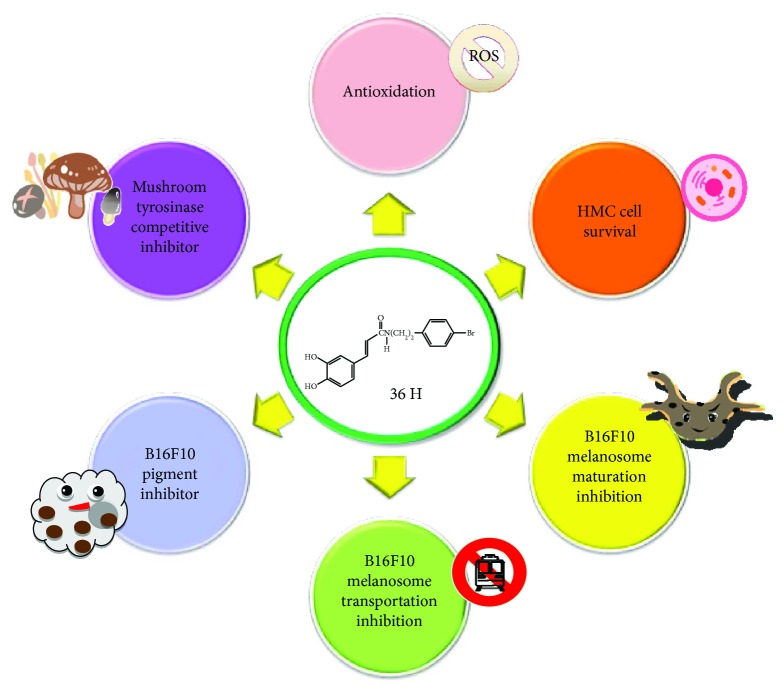
Proposed schematic diagram of compound 36H biofunctions.

Human skin melanin is driven by the intercellular movement of melanin-containing melanosomes from the extremities of HMC dendrites to neighboring keratinocytes. When it is carried by the actin filament, melanosome moves to the dendritic tail section, through exocytosis, and is transported into keratinocytes [26]. The greater the amount of melanin that is transferred into keratinocytes, the darker is the color of the skin [27]. The movement on the microtubule depends on the dynein-dynactin motor complex. Mreg forms a complex with Rab-interacting lysosomal protein and p150(Glued) which is a subunit of dynactin [28]. Mreg adjusts a shedding system which transports melanosome from HMC to keratinocytes. The shedding process from HMC of melanosome-rich packages undergoes the phagocytosis of keratinocytes. The shedding not only takes place principally at dendritic extremities but also around the center areas, having adhesion to keratinocytes, tightening behind the forming packages, and apparent self-abscissions [29]. The movement on the actin filament requires Myo5a, Rab27a, and MLPH as the connecting bridge [30]. 36H downregulated the protein expression for Myo5a and might prevent a darkening of skin color. Collectively, the data shows that 36H is an effective skin-whitening agent that has the potential for cosmetic applications (Figure 7).

## Figures and Tables

**Table 1:** The nucleotide sequences of primers used in this study.

Tyrosinase	
Forward: 5′-CTGCCAACGATCCTATCTTCCT-3′	
Reverse: 5′-GGTTATGTCCAATGGGTGCATT-3′	
MITF	
Forward: 5′-TTGGTGCCACCTAAAACATTGT-3′	
Reverse: 5′-CCGTTGGGCTTGCTGTATG-3′	
TRP-1	
Forward: 5′-GGTTTATTTGACACGCCTCCTT-3′	
Reverse: 5′-AGACTTCGAACAGCAGGGTCAT-3′	
DCT	
Forward: 5′-GTTCCTTTCTTCCCTCCAGTGACTA-3′	
Reverse: 5′-GTGGGCCAACCTGGAGTTT-3′	
Pmel17	
Forward: 5′-GGATGGTACAGCCACCTTAAGG-3′	
Reverse: 5′-CAGGATCTCGGCACTTTCAATAC-3′	
Rab27a	
Forward: 5′-CAGGGAAAAAAGAGTGGTGTACAGA-3′	
Reverse: 5′-ACGCTGTCGTTAAGCTACGAAAC-3′	
Myo5a	
Forward: 5′-GCCCAGATTGTGAAAGTGTTGA-3′	
Reverse: 5′-CCTGTCTCGTAAACGCATCTGT-3′	
MLPH	
Forward: 5′-AAGAGACCAGAGGACCCAAATG-3′	
Reverse: 5′-TTTCCGATCAAAAGAATCATCATC-3′	
Mreg	
Forward: 5′-TGGTGAGGGATGATGAGAAGAAT-3′	
Reverse: 5′-TCTGCCACTCCTCTGAGTCTTTG-3′	

**Table 2:** DPPH free radical-scavenging property, reducing power, and chelating ability of 36H at different concentrations (10, 50, and 100 *μ*M) are presented. The concentration of three specific positive controls, vitamin C, BHA, and EDTA, is 100 *μ*M. The data is representative of 3 experiments as mean value ± SD.

Samples	DPPH inhibition (%)	Ferric-reducing power (OD value)	Chelating capacity (%)
Vitamin C (100 *μ*M)	85.3 ± 6.2	—	—
BHA (100 *μ*M)	—	0.95 ± 0.07	—
EDTA (100 *μ*M)	—	—	80.2 ± 0.4
36H (10 *μ*M)	<10.0	0.19 ± 0.01	<10.0
36H (50 *μ*M)	25.8 ± 5.1	0.42 ± 0.04	13.1 ± 2.3
36H (100 *μ*M)	60.4 ± 2.8	0.85 ± 0.07	43.2 ± 3.6
